# Supplementary material for: MetaRibo-Seq measures translation in microbiomes
Source: Nat Commun. 2020 Jun 29;11:3268. doi: 10.1038/s41467-020-17081-z (PMC7324362; doi:10.1038/s41467-020-17081-z)
Supplement: Supplementary file 10 — Supplementary Data 7 [file 41467_2020_17081_MOESM10_ESM.zip › File2/Confidence_High_Taxonomy/15223_out.krona.html]

Javascript must be enabled to view this page.

members
magnitude
magnitudeUnassigned
count
unassigned
taxon
rank

15223\_out

8

2
8
superkingdom

976
8
phylum

200643
class
8

8
order
171549

815
8
family

8
genus
816

457395

SRS058770\_contig\_number\_13475
1
species

4
species
821

SRS105153\_contig\_number\_24631SRS142503\_contig\_number\_16255SRS143085\_contig\_number\_1077SRS893288\_contig\_number\_584

species
1

SRS142542\_contig\_number\_20345
28116

1739517

SRS054905\_contig\_number\_13890SRS145308\_contig\_number\_4177
2
species
